# Impact of emerging compound droughts on forests: A water supply and demand perspective

**DOI:** 10.1111/plb.70080

**Published:** 2025-08-11

**Authors:** C. Werner, M. Bahn, T. E. E. Grams, C. Grossiord, S. Haberstroh, G. Lenczner, D. Tuia, H. Vallicrosa

**Affiliations:** ^1^ Ecosystem Physiology UNR, University Freiburg Freiburg Germany; ^2^ Department of Ecology University Innsbruck Innsbruck Austria; ^3^ Ecophysiology of Plants, Land Surface—Atmosphere Interactions School of Life Sciences, Technical University of Munich Freising Germany; ^4^ Plant Ecology Research Laboratory PERL School of Architecture, Civil and Environmental Engineering, EPFL Lausanne Switzerland; ^5^ Community Ecology Unit Swiss Federal Institute for Forest, Snow and Landscape WSL Lausanne Switzerland; ^6^ Environmental Computational Science and Earth Observation Laboratory ECEO School of Architecture, Civil and Environmental Engineering, EPFL Sion Switzerland

**Keywords:** compound droughts, ecophysiological regulation, forest, hydraulic regulation

## Abstract

The intensification of climate change‐induced drought results in unprecedented tree and forest die‐offs worldwide, increasingly driven by compound droughts. In this review, we examine the impacts of emerging compound droughts, which involve co‐occurring stressors like soil drought and high temperature, along with elevated vapour pressure deficit over prolonged periods and at higher frequency. We explore the physiological and ecological mechanisms underlying tree water and carbon regulation during these extreme conditions, focusing on the balance between water demand and supply, the role of acclimation, and its consequences for ecosystem‐level functions. By examining the mechanisms at play from the organ to the ecosystem‐scale, we provide a comprehensive understanding of how trees and forests are likely to respond to an increasingly unpredictable climate with a higher likelihood of compound droughts.

## Graphical abstract

This review examines the physiological and ecological responses of trees to emerging compound droughts from a water demand and supply perspective, as well as the role of acclimation and consequences for ecosystem‐level functions.

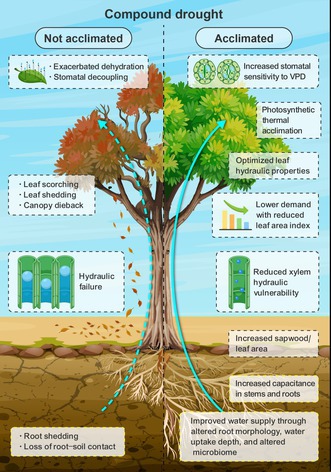

## Introduction

The impacts of climate change‐induced drought are intensifying across ecosystems worldwide, resulting in unprecedented die‐offs of trees and entire forests (Anderegg *et al*. 2013c; Allen *et al*. 2015; Hartmann *et al*. 2022). This trend is fuelled not only by more frequent and severe droughts, but also by an increase in compound climatic events comprising several stressors, whose occurrence is likely to accelerate in the future (e.g. Bevacqua *et al*. 2022).

‘Compound droughts’ refers to exceptional drought conditions characterized by co‐occurring stressors (i.e. air and soil dryness, together with extreme heat), increased intensity (i.e. drier and hotter conditions), extended duration and/or higher frequency (Hao *et al*. 2022), setting them apart from historical patterns, therefore making them even harder to predict or manage. Key factors include the co‐occurrence of soil drought (driven by a lack of precipitation) alongside excessively high air temperatures (Zscheischler & Seneviratne 2017; Seneviratne *et al*. 2021; Hammond *et al*. 2022), and a rise in evaporative demand, driven by higher vapour pressure deficit (VPD) (Grossiord *et al*. 2020; McDowell *et al*. 2022; Novick *et al*. 2024). Emerging compound droughts disrupt ecosystems even when the amount of annual rainfall does not change, as rainfall patterns shift towards more intense but infrequent events, creating longer and repeated dry periods, with significant ecological impacts (Feldman *et al*. 2024).

During compound droughts, heightened VPD intensifies drought stress on vegetation by accelerating soil moisture loss and plant water demand (Penman 1948; Massmann *et al*. 2019), creating a new form of atmospheric drought, which often coincides with soil droughts, but can also precede soil drying. Furthermore, elevated air temperatures accelerate the heating of vegetation surfaces, particularly if coupled with low soil moisture (e.g. Still *et al*. 2023; Gauthey *et al*. 2024), which may approach critical limits for key physiological processes (O'Sullivan *et al*. 2017). For example, compound droughts have been identified to explain 46% of tree mortality across Europe (Gazol & Camarero 2022).

As the frequency and recurrence of compound droughts have increased globally (Seneviratne *et al*. 2021; Hao *et al*. 2022; Calvin *et al*. 2023), ecosystem recovery times from extreme events have also increased (Schwalm *et al*. 2017; Zhang *et al*. 2021), potentially preventing full recovery before the following extreme event occurs (Schwalm *et al*. 2017; Seneviratne & Ciais 2017). This can ultimately lead to severe drought legacy effects, which can alter ecosystem responses to subsequent drought. These legacy effects can be both negative (e.g. retarded recovery) and positive (e.g. through acclimation responses), thereby affecting tree and ecosystem responses to recurrent droughts (Müller & Bahn 2022). Thus, the frequency and recurrence of compound droughts add an additional dimension of impact, potentially affecting ecosystem resilience.

Trees manage water fluxes across the entire soil–plant–atmosphere continuum, finely tuning their physiological responses to balance the supply and demand of water. In this context, we define plant *water demand* as the amount of water needed for sustaining physiological processes, such as transpiration, CO_2_ uptake, and growth. In contrast, *water supply* refers to the availability of water in the plant's surrounding environment. The balance between the water demand and supply determines the plant water status. Hydraulic capacitance, i.e. water that can be released from storage tissues such as tree trunks (Goldstein *et al*. 1998; Meinzer *et al*. 2008; Jupa *et al*. 2016), can further help to buffer the diurnal and seasonal balance of water supply and demand. This coordination is crucial, as it enables trees to cope with fluctuations in soil water availability and atmospheric conditions to avoid critical thresholds of non‐return. Examining how trees manage these interconnected constraints provides new insights into their resilience mechanisms under extreme conditions, helping us to better predict ecosystem responses to compound drought events, and potentially informing adaptive forest management practices to mitigate drought impacts in a warming world.

In the following, we will present a holistic overview of the evidence pertaining to emerging compound droughts (Section 2), and discuss their impact on the organ, whole tree, and ecosystem scale from a water demand (Section 3) and supply (Section 4) perspective, including the potential mitigating role of acclimation to these conditions (Section 5), and its feedback to ecosystem‐scale processes (Section 6).

We will not focus on the mechanisms that induce tree mortality, as these have been covered extensively elsewhere (Allen *et al*. 2015; Anderegg *et al*. 2013b; Hartmann *et al*. 2022; McDowell *et al*. 2022; McDowell & Allen 2015).

## EMERGING EVIDENCE OF COMPOUND DROUGHTS

**Fig. 1:**
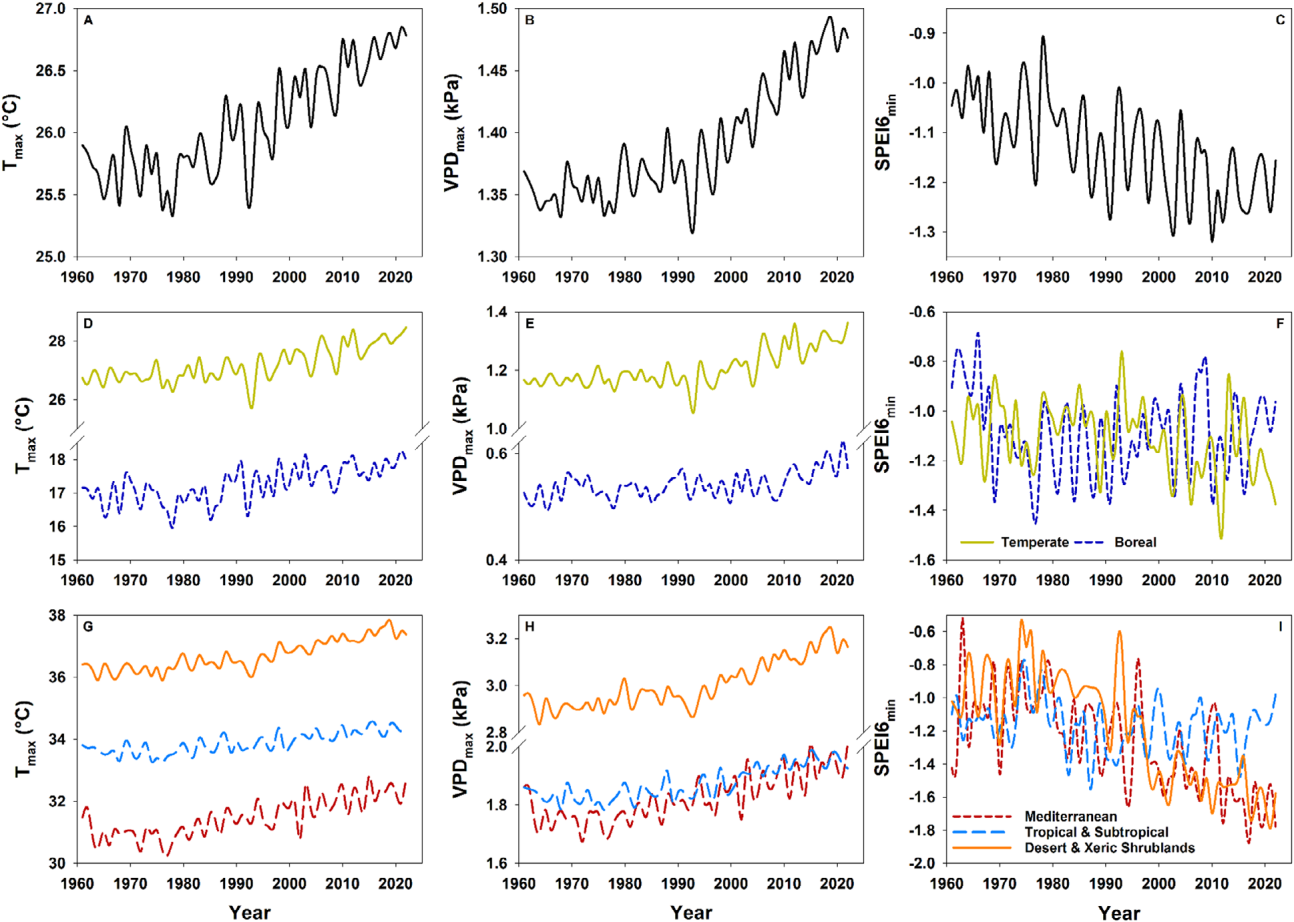
Maximum monthly air temperature (*T*_max_), maximum monthly vapour pressure deficit (VPD_max_), and minimum annual standardized precipitation evapotranspiration index (SPEI6_min_) calculated over the time span of 6 months per year at the global scale (A–C), and for specific biomes: (D–F) Boreal and Temperate forests; (G–I) Mediterranean, Deserts and xeric shrubland, and Tropical and subtropical biomes (G–I). Data for *T*_max_ and VPD_max_ computation were derived from the Climatic Research Unit (CRU v. 4.08; University of East Anglia) and NCAS (Harris *et al*. 2014, 2020). Data for SPEI computation was derived from the global SPEI database (v. 2.9) (Beguería *et al*. 2010a, 2014; Vicente‐Serrano *et al*. 2010a). For detailed calculations, see Appendix A.

Globally, a significant and consistent increase in maximum air temperature (*T*_max_), maximum vapour pressure deficit (VPD_max_), and drought severity, as depicted by decreasing minimum annual Standardized Precipitation Evapotranspiration Index, calculated over a time span of 6 months (SPEI6_min_), has been observed between 1961 and 2022 (Fig. 1A–C). These trends are strongly reflected in major global biomes, including the temperate zone, the Mediterranean, and deserts and xeric shrublands (Fig. 1D–I). The latter two areas experienced a particularly strong increase in drought severity compared to global patterns (Fig. 1F). In boreal, tropical and subtropical zones, only *T*_max_ and VPD_max_ increased significantly, while no clear trend was evident in terms of drought severity. These patterns align with other studies reporting increasing global atmospheric aridity (Yuan *et al*. 2019; Fang *et al*. 2022) and a rise in the number, days and intensity of heatwaves (Dunn *et al*. 2024a), with negative impacts on vegetation greenness, gross primary and net ecosystem productivity (Yuan *et al*. 2019; He *et al*. 2022). In contrast, previously reported global trends in SPEI on a 12‐month basis were less clear and driven mainly by an increased atmospheric evaporative demand (Seneviratne *et al*. 2021; Vicente‐Serrano *et al*. 2022; Dunn *et al*. 2024a). However, some regions, such as Western and South Africa, East Asia, the Mediterranean Basin or Central Europe, also show decreasing SPEI12 and, thus, an increase in soil drought severity (Seneviratne *et al*. 2021; Dunn *et al*. 2024a). Forest ecosystems from different biomes respond to different drought periods, ranging from 12 to 14 months for semi‐arid to 3–5 months for cold and more humid sites (Vicente‐Serrano *et al*. 2014). As forests in humid temperate locations respond most strongly to a drought period of 6–8 months (Vicente‐Serrano *et al*. 2014; Haberstroh & Werner 2022), in the following, we use SPEI calculated over 6 months as the most meaningful ecological drought index for humid forests of the temperate zone.

**Fig. 2:**
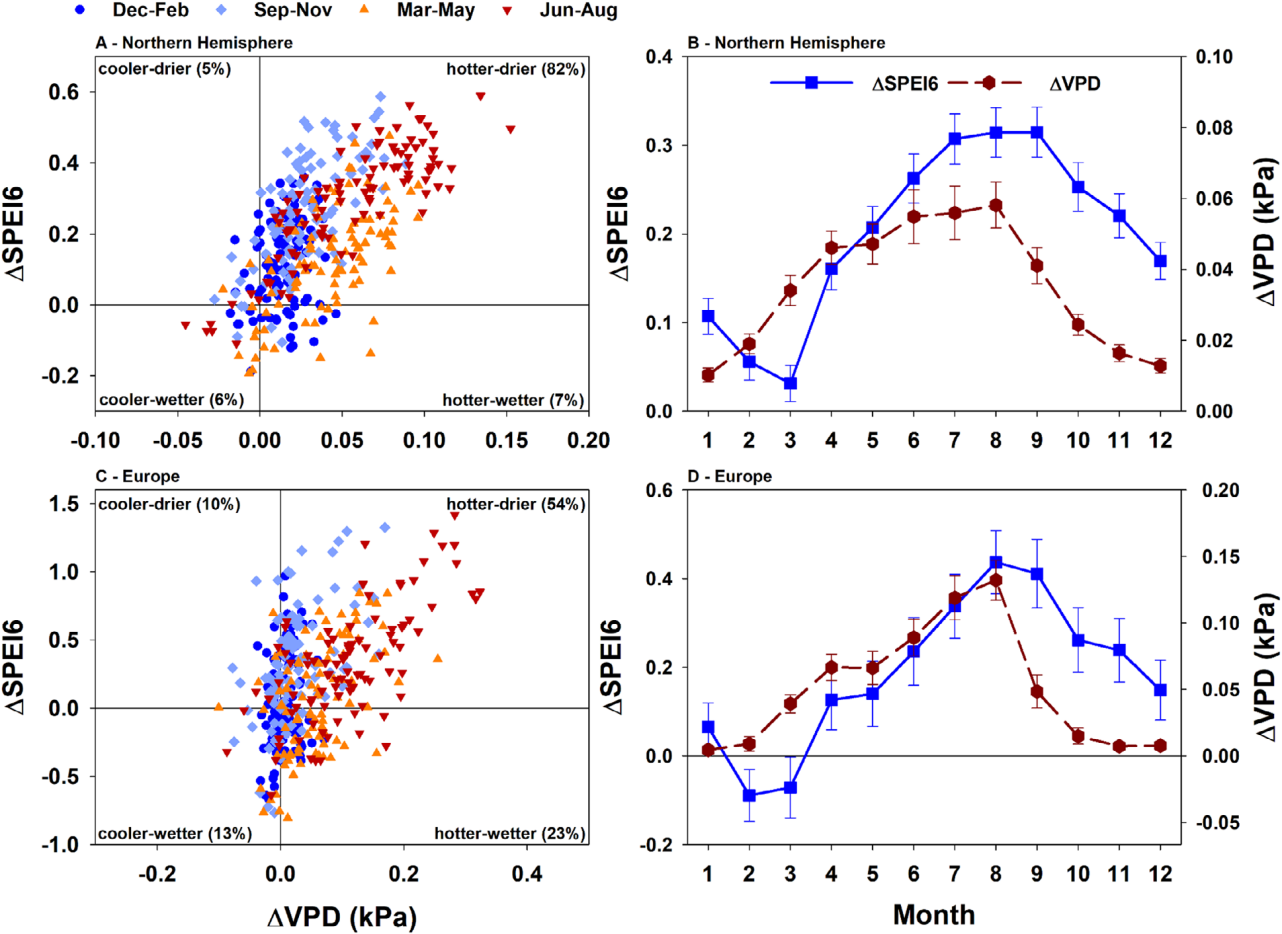
Monthly (A, C) and average deviation ±1SE (B, D) of monthly mean vapour pressure deficit (ΔVPD) and monthly standardized precipitation evapotranspiration index (ΔSPEI6) for the entire Northern Hemisphere (A, B) and for Europe (C, D). Data from 1991 to 2022 are shown as deviations from the standard reference period 1961–1990. Percentages labelled *cooler‐drier, cooler‐wetter, hotter‐drier* and *hotter‐wetter* correspond to the number of months falling into each category (in comparison to the standard reference period 1961–1990). Data for VPD computation were derived from the Climatic Research Unit (CRU v. 4.08; University of East Anglia) and NCAS (Harris *et al*. 2014, 2020). Data for SPEI computation was derived from the global SPEI database (v. 2.9) (Beguería *et al*. 2010a, 2014; Vicente‐Serrano *et al*. 2010a). For detailed calculations see Appendix A2. Please note the different scales between panels.

The following patterns are clearly evident in the analysed datasets: from 1991 to 2022, 82% and 54% of all months in the Northern Hemisphere and in Europe, respectively, were hotter and drier than the long‐term average (1961–1990; Fig. 2A,C). Only 6% (Northern Hemisphere; Fig. 2A,B) and 13% (Europe; Fig. 2C,D) were colder and wetter. It is noteworthy that the strongest increase in both, VPD and drought severity occurred in the summer months (July–September; Fig. 2B,D), with a peak in August. These data underline exceptional drought and heat occurrence in recent years, especially in Central Europe (Hari *et al*. 2020; Ionita & Nagavciuc 2021; Moravec *et al*. 2021; Knutzen *et al*. 2025). The 2018–2022 drought in Central Europe set a new benchmark for drought duration and extent, with an air temperature anomaly of +2.8 K (Rakovec *et al*. 2022). Moreover, 2018–2022 was characterized by persistent hot droughts in Central Europe (Knutzen *et al*. 2025). This clearly indicates the increase in severity, frequency and duration of compound events in Central Europe, which are projected to increase further in the future (Bevacqua *et al*. 2022; Calvin *et al*. 2023; Luca & de Luca & Donat 2023; Rakovec *et al*. 2022; Seneviratne *et al*. 2021).

## FACTORS REGULATING WATER DEMAND AND FEEDBACK ENHANCING COMPOUND DROUGHTS AT THE LEAF‐TO‐TREE LEVEL

**Fig. 3:**
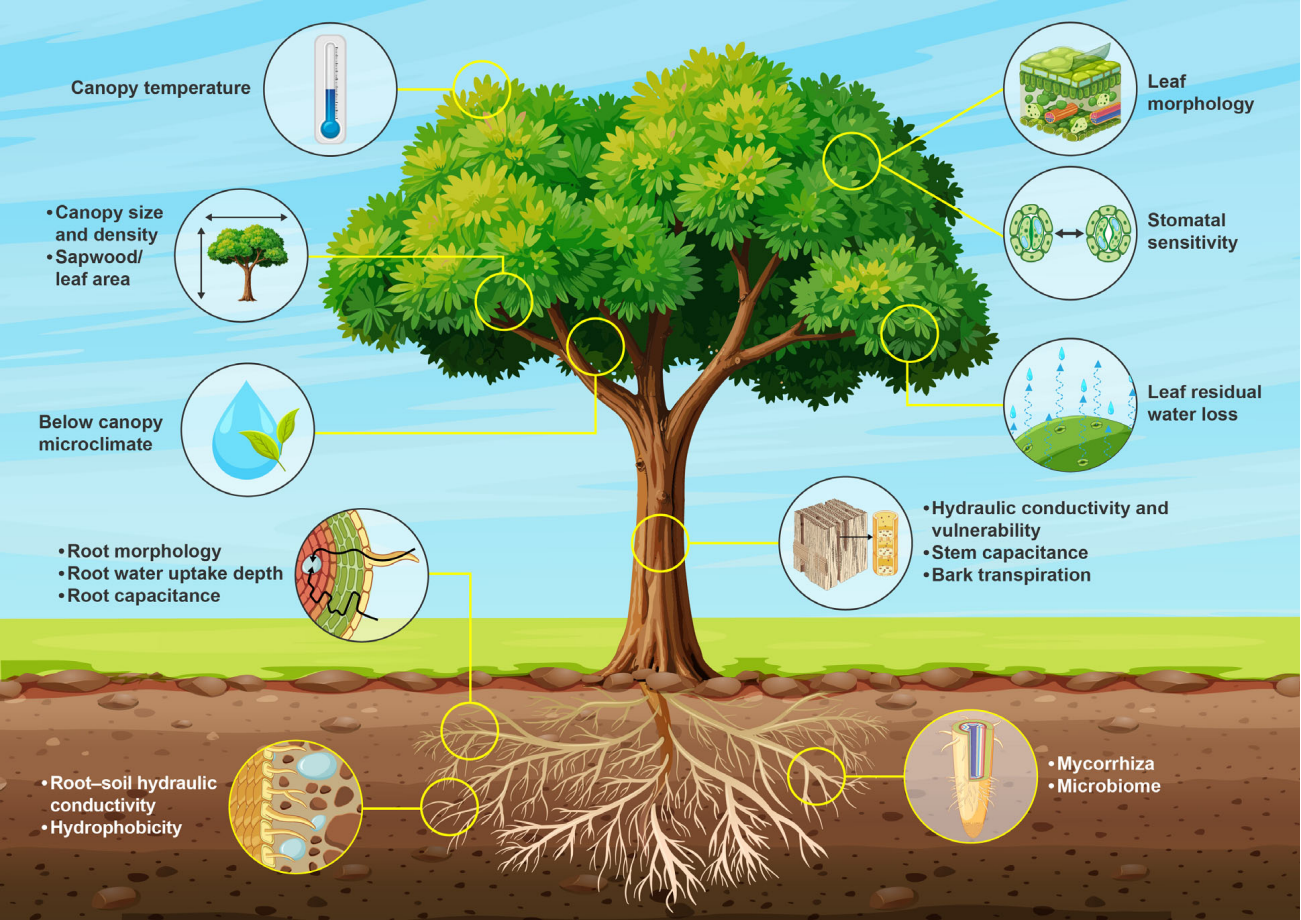
Key structural and physiological traits and processes regulating tree water fluxes along the soil–plant–atmosphere continuum and affecting water supply and demand.

Compound droughts can lead to tree water demand exceeding supply, inducing reduced photosynthesis, impaired growth, leaf wilting, increased susceptibility to pests and pathogens, stomatal closure and, if prolonged, potential tissue damage, hydraulic failure, or death (e.g. Centritto *et al*. 2011; Hammond *et al*. 2022; Bastos *et al*. 2023; Laoué *et al*. 2024; Haberstroh *et al*. 2026, this issue). The tree water demand is deterimed by the transpirational area and abiotic environmental factors (e.g. temperature, light, wind, VPD) and is regulated by various internal mechanisms at the leaf‐to‐whole‐tree scale (Fig. 3).

The regulation of water demand starts with fine‐tuned stomatal responses to environmental drivers and enables both the optimization of carbon gain and the control of water loss (Cowan *et al*. 1977; Farquhar & Sharkey 1982; Ball *et al*. 1987). While ample water supply optimizes carbon gain, a lack of sufficient water supply to fulfil the demand induces water‐saving strategies that constrains carbon gain (Ball *et al*. 1987; Centritto *et al*. 2011; Medlyn *et al*. 2011). In such instances, stomatal closure efficiently prevents detrimental dehydration, runaway cavitation and hydraulic failure (Wolf *et al*. 2016; Anderegg *et al*. 2018b). Stomata respond to cues like irradiance, CO_2_ concentration, VPD, leaf water potential and hormonal as well as electrical signals (Buckley 2019). The stomatal sensitivity to environmental drivers (i.e. the relative decrease in stomatal conductance (g_s_) with a given driver) varies widely among species (Grossiord *et al*. 2020). Isohydric species (i.e. species that tend to close their stomata rapidly during dry periods) demonstrate stricter water loss control as soil moisture drops and VPD rises than anisohydric species (i.e. species that maintain stomata open for longer periods as the soil dries out) (Tardieu & Simonneau 1998). Trees can adapt their degree of isohydricity to prevailing environmental constraints (e.g. Feng *et al*. 2019; Haberstroh *et al*. 2022a). Typically, plants show coordination in stomatal traits across broad environmental gradients to optimize water management and carbon gain (Xie *et al*. 2022), with lower maximum g_s_, and morphological traits preventing water loss, including smaller and narrower stomata (leading to lower maximum g_s_) in drier areas compared to wetter ones, leading to higher water use efficiency (e.g. Saurer *et al*. 2014). Additionally, higher nocturnal temperatures and VPD can result in substantial nocturnal water losses (e.g. Zeppel *et al*. 2014; Resco de Dios *et al*. 2019; Chowdhury *et al*. 2022).

During the day, despite stomatal closure, higher VPD drives increased plant transpiration (Grossiord *et al*. 2020), accelerating water loss during compound droughts. Moreover, water loss continues after stomatal closure through the cuticle and incomplete stomatal closure (i.e. the minimum stomatal conductance (g_min_; e.g. Duursma *et al*. 2019)) and bark transpiration (e.g. Lintunen *et al*. 2021). Hence, plants with high g_min_ and bark transpiration lose more water, accelerating internal water depletion and eventually leading to a loss of hydraulic conductivity, potentially inducing hydraulic failure through embolism (Brodribb *et al*. 2021; Cochard 2021; Wang *et al*. 2024). Stem capacitance and dynamic supply of sap flow from different sapwood depths (Dumberger *et al*. 2025) may help buffer this accelerated dehydration and delay leaf wilting (Schymanski *et al*. 2013). Stem water supply for transpiration differs between species but can contribute 5% to 50% of the daily water budget (Goldstein *et al*. 1998; Meinzer *et al*. 2004; Oliva Carrasco *et al*. 2015; Dietrich *et al*. 2018; Ziemińska *et al*. 2020). The tree water deficit is often measured in diurnal changes of stem swelling and shrinkage (Donfack *et al*. 2026, this issue), reflecting the water status of trees (Dietrich *et al*. 2018), and is generally enhanced under drought (Kinzinger *et al*. 2024). The fact that some tree species prioritize trunk refilling after severe droughts over transpiration may further highlight the importance of capacitance for drought survival (Kühnhammer *et al*. 2023).

**Fig. 4:**
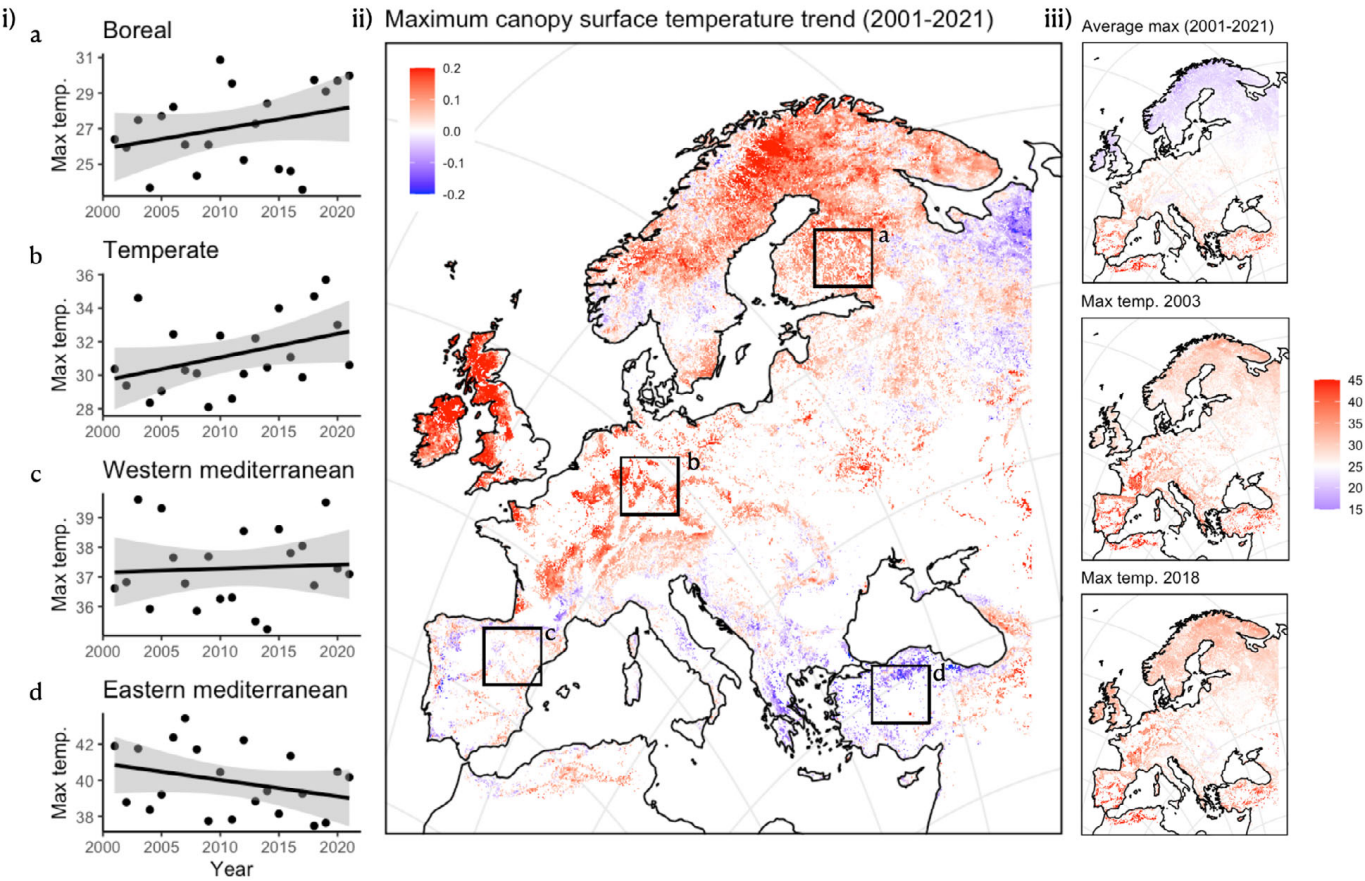
Canopy surface temperature trends (from remote sensing 2001–2021 data). White regions correspond to areas without dense vegetation. (i) Linear regression fits (± SD) for the maximum monthly canopy surface temperature per year. (ii) Maximum canopy surface temperature trend per pixel at 5 km resolution. Blue colours describe negative trends and red colours describe positive trends. (iii) Average maximum canopy surface temperature, maximum canopy temperature of 2003, and maximum canopy surface temperature of 2018. 20°C is set as white on the scale bar, with blue <20°C and red >20°C.

Nevertheless, higher foliar temperatures accelerate plant desiccation, amplifying tree mortality during compound droughts (Cochard 2021). Indeed, as exemplified for Europe (Fig. 4), maximum canopy surface temperature has significantly increased during the last two decades. This general trend is seen throughout the continent, with northern Europe experiencing the greatest warming (e.g. Norway, Finland, Sweden, United Kingdom and Ireland). In contrast, regions with the highest canopy surface temperature today (i.e. the entire Mediterranean Basin) show little to no warming trend. Whether this trend is driven by a higher thermoregulation capacity in plants from these regions or by ongoing acclimation processes improving temperature regulation (e.g. shifts in canopy size modifying sensible heat flux; Gauthey *et al*. 2023; Muller *et al*. 2021, 2023) is unknown. Still, an overall chronic canopy temperature rise is partially due to the increased frequency of extreme events, leading to exacerbated overheating. For instance, during two recent compound events (i.e. 2003 and 2018), the maximum canopy temperature reached up to 45°C in regions rarely exposed to such extremes (e.g. central France, Germany; Fig. 4). Those high temperatures enhance the water demand while increasing the risk of approaching critical thermal limits of leaf functioning. Such high temperatures induce rapid heat‐stress damage to the photosystems, protein degradation, and irreversible leaf scorching, as already observed under recent extreme events, even in temperate forests (e.g. Miranda *et al*. 2020; Kunert *et al*. 2021; Still *et al*. 2023). Similarly, tropical forests have been predicted to approach these thermal limits under extreme heatwaves (Doughty *et al*. 2023; but see Winter 2024).

During compound droughts, low soil moisture should induce reduced transpirational cooling despite elevated VPD, exacerbating the heat load on the leaf and thus the leaf‐to‐air VPD (e.g. Gauthey *et al*. 2024; Posch *et al*. 2024). In this context, more and more studies report ‘stomatal decoupling’, whereby high temperatures induce stomatal opening (e.g. Drake *et al*. 2018; Diao *et al*. 2024; Gauthey *et al*. 2024). The exact mechanisms triggering this response are largely unknown (Mills *et al*. 2024), and their importance for evaporative cooling remains to be tested. Furthermore, drought‐induced stomatal closure presents a challenge for controlled dissipation of absorbed solar energy in the photosystems, when the Calvin cycle is downregulated due to a lack of CO_2_ (Martinez‐Ferri *et al*. 2000). Although several mechanisms are in place to facilitate the controlled dissipation of excess light energy in the photosynthetic antenna, such as the xanthophyll cycle, D1‐protein turnover of photosystem II, the Mehler reaction and reactive oxygen species (ROS)‐scavenging enzymes, photoinhibition can result in further carbon losses (Werner *et al*. 2001; Hikosaka 2021) and leaf bleaching, thereby exacerbating the stress effects on plants. Moreover, elevated temperatures will favour photorespiration, and a low CO_2_/O_2_ ratio during stomatal closure will further amplify this effect. Higher temperatures can also induce the transient emission of secondary compounds, such as volatile organic compounds (VOCs), as protection against heat and excess radiation (Rennenberg *et al*. 2006; Holopainen & Gershenzon 2010; Loreto & Schnitzler 2010; Jud *et al*. 2016; Werner *et al*. 2020). For example, isoprene can act as a signalling molecule, altering gene expression and metabolomics to increase heat tolerance, for example, by stabilizing thylakoid membranes or scavenging of ROS (Velikova *et al*. 2012; Harvey *et al*. 2015; Lantz *et al*. 2019; Monson *et al*. 2021; Bergman *et al*. 2025). However, under severe heat stress, particularly when photosynthesis is strongly inhibited, the biosynthesis of VOCs, especially those produced in the chloroplast, such as isoprene, can also be significantly reduced (Loreto & Schnitzler 2010; Fares *et al*. 2011; Yáñez‐Serrano *et al*. 2019; Werner *et al*. 2020). Under prolonged hot droughts species adjust carbon allocation into VOCs (Kreuzwieser *et al*. 2021; Ladd *et al*. 2023), but it has also been shown that drought can exert an overriding effect on isoprene emission, potentially offsetting the increase in VOC emissions due to rising temperatures (Fortunati *et al*. 2008; Centritto *et al*. 2011). Higher temperatures do further enhance the volatility of VOCs from storage organs, for example, in conifers, with cascading effects on atmospheric processes due to the high reactivity of VOCs with atmospheric chemistry (Makkonen *et al*. 2012; Guenther 2013). Moreover, constitutive or induced VOC emissions have a protective function against biotic stress, as they serve as defence compounds against insects or pathogen attacks (Holopainen & Gershenzon 2010), which may increase under extreme stress. While many plant species in hot and dry climates have highly efficient protection mechanisms, it remains largely unknown to what extent species of temperate or boreal climates, which generally have a lower capacity to dissipate excess radiation, may adapt. Therefore, even before reaching critical temperature limits for plant survival, significant impacts on photosynthetic and leaf regulatory processes can be anticipated under compound droughts.

Changes in the water demand during compound droughts will also be driven by changes in evaporative surfaces (i.e. total leaf area). In particular, leaf morphological and functional traits determine drought sensitivity (Kunert *et al*. 2026, this issue; Kretz *et al*. 2026, this issue). Excess heat combined with drought often triggers leaf shedding and scorching, minimizing water loss. However, this diminishes photosynthetic capacity and growth, ultimately weakening trees and potentially increasing the risk of carbon starvation (e.g. McDowell & Sevanto 2010; McDowell *et al*. 2022). Carbon starvation and hydraulic dysfunction often co‐occur, further weakening the trees, enhancing the risk of tree mortality (Alderotti *et al*. 2024; Trueba *et al*. 2024). Early leaf shedding can be a strategy to reduce transpiration and prevent hydraulic failure (Bréda *et al*. 2006), particularly in semi‐deciduous species adapted to regular seasonal droughts (e.g. Werner *et al*. 1999). However, recent studies indicate that in temperate trees, this is more likely a direct consequence of hydraulic failure (Walthert *et al*. 2021; Arend *et al*. 2022). The higher frequency of compound droughts further reduces growth. It can alter carbon allocation, resulting in less dense canopies over time (e.g. Gauthey *et al*. 2023; Mas *et al*. 2026, this issue) with increased root/shoot ratios and, thus, shifts the supply‐to‐demand balance. These morphological responses may help trees to better withstand repeated drought and thus reduce future drought stress (Hikino *et al*. 2026, this issue; see also Section 5).

However, legacy effects resulting in retarded recovery from prior extreme events, for example, due to reduced hydraulic capacity or depleted carbon and nutrient reserves, can render trees vulnerable during recurrent droughts (Kannenberg *et al*. 2019; Oberleitner *et al*. 2022). Drought legacy is strongly modulated by the environmental conditions preceding and following severe droughts (Pohl *et al*. 2023; Heinrich *et al*. 2026, this issue; Ruehr & Nadal‐Sala 2026, this issue). While drought stress memory may enhance physiological resilience to subsequent events (Godwin & Farrona 2020), a high frequency of extreme events can weaken trees, rendering them vulnerable to storms and windthrow, pests and diseases, ultimately resulting in tree mortality (Choat *et al*. 2018; McDowell *et al*. 2022). Moreover, drought‐induced leaf shedding and increased tree mortality can result in changes in local microclimates (Anderegg *et al*. 2013b; Zellweger *et al*. 2020), leading to reduced shade and increased soil dryness, further stressing trees by creating hotter, drier conditions (see also Section 6). However, many uncertainties remain regarding the species‐specific variability in canopy shifts over time, as well as about their rates and consequences for tree water demand during compound droughts.

## FACTORS REGULATING WATER SUPPLY DURING COMPOUND DROUGHTS AT TREE LEVEL

As detailed above, compound drought intensifies tree water use, thereby reducing soil water availability for the trees. At the same time, elevated temperature and VPD directly affect water supply by increasing evaporation from the soil surface, reducing soil moisture and water availability, especially when open tree canopies allow for high energy input (Anderegg *et al*. 2013b). In addition, there is emerging evidence that increasing drought frequency and intensity can significantly alter a range of ecohydrological factors relevant to plant water supply. For instance, 5 years of recurrent experimental summer drought in a beech/spruce forest resulted in increased hydrophobicity of the forest soil, with almost five times higher repellence (Grams *et al*. 2021). During subsequent slow rewetting, altered preferential water flow caused deeper soil layers to partially rewet faster than the hydrophobic topsoil. Indeed, drought history has been suggested to be more important than the actual antecedent soil moisture status regarding hydrophobicity and infiltration behaviour (Gimbel *et al*. 2016). Moreover, drought and increasing aridity typically reduce soil aggregate stability (Zhang *et al*. 2018; Berdugo *et al*. 2020) leading to a loss of macroaggregates and a significant decline in saturated hydraulic conductivity and total porosity (Zhang *et al*. 2018), and hence potential water storage. Furthermore, recurrent droughts have been found to reduce soil hydrological connectivity (Smith *et al*. 2017; Blaurock *et al*. 2021), with changes in pore size water mixing, thus decreasing plant water access and use (Radolinski *et al*. 2025). Generally, the critical threshold of water limitation in the plant–soil continuum is further shaped by soil texture through differences in soil hydraulic conductivity between sandy and fine‐textured (e.g. clay) soils, which feeds back on the plant sensitivity to VPD (Wankmüller *et al*. 2024).

Beyond soil factors, a range of plant factors determine the water supply of trees and are particularly relevant for tree resilience in the face of compound droughts. On a larger scale, subsurface hydrological processes mediate tree vulnerability to extreme climatic drought. However, the effects are highly species‐specific (McLaughlin *et al*. 2020), leading to a distinct ecohydrological niche separation among species as precipitation is decoupled from water availability (Chitra‐Tarak *et al*. 2018, 2021; Ding *et al*. 2021). Detailed model analyses suggest that, next to soil hydraulic conductivity, root distribution is key to predicting and interpreting transpiration reductions during drought (Carminati & Javaux 2020). The depth of water absorption is primarily dependent on the availability of water: as shallow soils dry out, water absorption is shifted to deeper soil layers, provided that roots are present. Indeed, trees can rapidly adjust their root water uptake depth within hours to days, even in response to a single rain event (Kinzinger *et al*. 2025). Moreover, in forest ecosystems with shallow soils overlying bedrock, rock moisture can provide a significant water reservoir for trees with deep sinker roots (Nardini *et al*. 2024). Thus, rooting depth is a key structural–morphological trait determining how quickly different trees approach hydraulic vulnerability thresholds during a drought event (Brinkmann *et al*. 2019; Kahmen *et al*. 2022). While deep taproots act as lifelines, they contribute little to total plant water uptake (Kühnhammer *et al*. 2023; Bachofen *et al*. 2024; Hackmann *et al*. 2026, this issue). However, the deeper part of the root system can have a significant impact on tree survival by increasing soil moisture in the upper layer through hydraulic redistribution (Prieto *et al*. 2012). This phenomenon is expected to increase in importance in extremely dry shallow soils under future climate conditions (Grünzweig *et al*. 2022; Hafner *et al*. 2026, this issue). It remains to be seen whether trees can increase their rooting depth under drought conditions and thus acclimate to more frequent droughts (Li *et al*. 2019; Mackay *et al*. 2020), which would also increase their ability to keep up with potentially declining groundwater levels.

Besides rooting depth, root branching patterns can also play an important role in water sourcing. Roots adapt their branching pattern to heterogeneous soil water conditions (Karlova *et al*. 2021) by linking changes in hydraulic flux with dynamic hormone redistribution (Mehra *et al*. 2022). Water uptake can also be optimized by increasing the proportion of younger roots, which are able to take up water more efficiently than older roots. Therefore, root shedding and localized formation of new roots represent an effective and flexible acclimation strategy of plants to a reduced water supply (Brunner *et al*. 2015; Nikolova *et al*. 2020; Zwetsloot & Bauerle 2021).

Mycorrhizae can also play an important role in the drought resistance and resilience of trees. While mycorrhizae increase the absorbing surface, mycorrhizal symbiosis also triggers numerous biochemical and physiological modifications in plants that enhance plant drought tolerance (Usman *et al*. 2021). Drought has been shown to increase root exudation and mycorrhizal symbiosis (Brunn *et al*. 2022; Lv *et al*. 2023). Drought resistance and resilience of tree communities depend, among others, on mycorrhizal association types (Sachsenmaier *et al*. 2024). Recently, it has also been suggested that, beyond mycorrhizal communities, the soil and even the leaf microbiome as a whole can alter drought tolerance of trees (Baldrian *et al*. 2023). There is evidence that the ectomycorrhizal fungal community composition responds to drought within a few months (Grams *et al*. 2021) and under successive summer droughts, reflecting the intensity of drought stress on the plant–soil system (Nickel *et al*. 2018; Weikl *et al*. 2023). However, the implications for water uptake and drought tolerance of trees are still unknown.

## WHOLE TREE ACCLIMATION

**Fig. 5:**
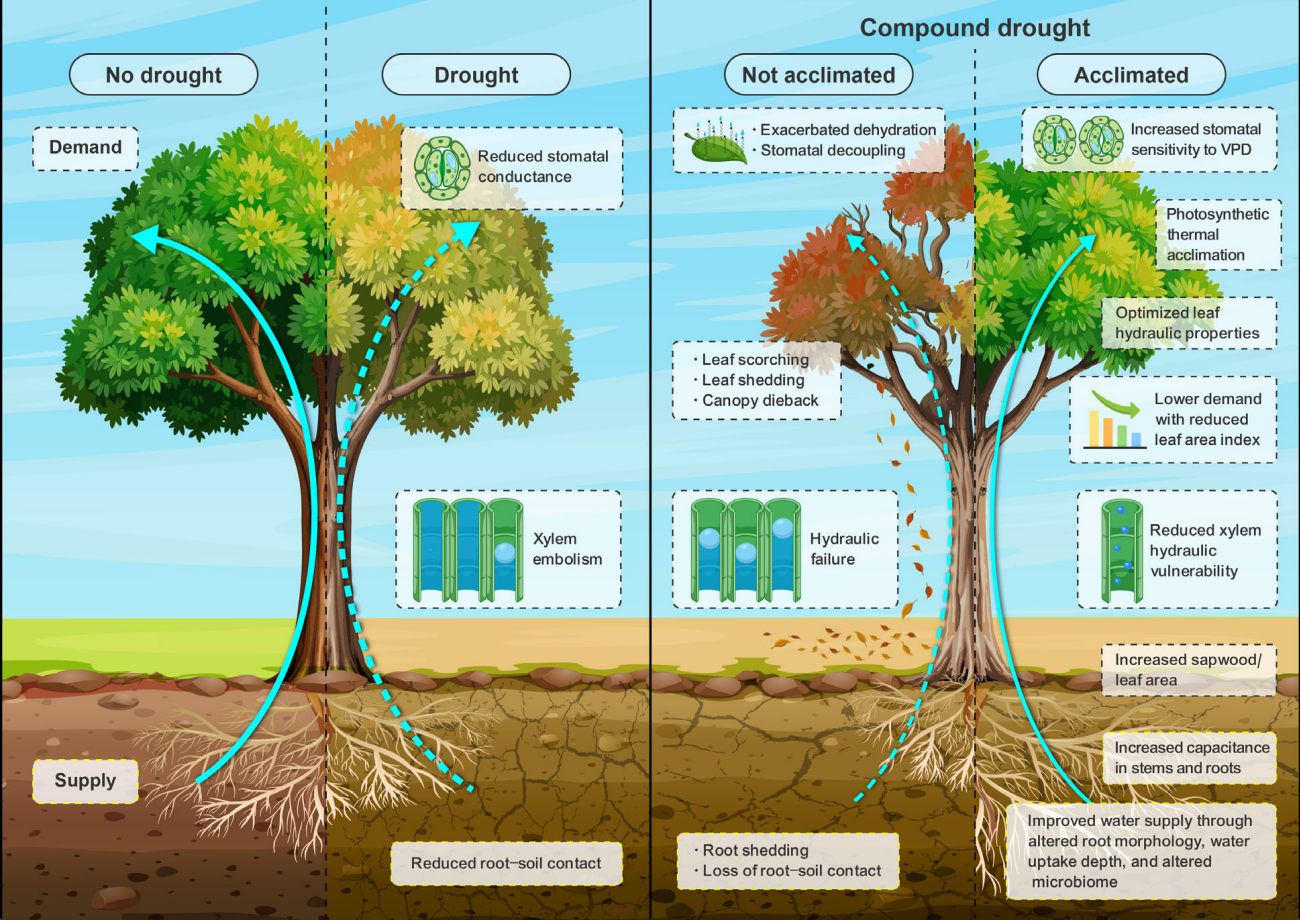
Water flux changes (arrows) and above‐ and belowground physiological, morphological and structural changes during favourable environmental conditions and in moderate drought (left panel) and compound drought and without or with acclimation (right panel).

Since compound droughts introduce compound stresses, as reduced water supply coincides with strongly increased water demand due to elevated temperatures and VPD, the extended duration of droughts may surpass the tree's ability to cope with these stressors. To persist under these conditions, trees must adjust their physiological and structural traits to avoid exacerbated drought effects, as prolonged exposure to compounding stressors can accelerate decline if recurrent droughts occur without acclimation (Fig. 5). Acclimation reflects longer‐term (weeks to decades) adjustments in plant biochemistry, physiology, or morpho‐anatomy that enhance or maintain plant ability to perform under novel environmental conditions, including developmental plasticity. As such, acclimation does not include the rapid biochemical and physiological changes triggered by sudden environmental fluctuations to prevent damage.

Acclimation to compound drought events should start at the stomatal level to allow plants to maintain optimal carbon assimilation while minimizing water loss (Marchin *et al*. 2016), thus avoiding hydrological failure. This process could occur by reducing stomatal sensitivity, increasing maximum stomatal conductance, and/or raising photosynthetic efficiency (e.g. the maximum carboxylation rate of photosynthesis and/or the maximum rate of electron transport; Centritto *et al*. 2011). Several studies have found evidence of increased stomatal sensitivity after prolonged exposure to elevated VPD, leading to increased transpiration and the maintenance of carbon assimilation (reviewed in López *et al*. 2021). In the long term, to avoid hydraulic impairments, plants would require simultaneous acclimation in hydraulic properties and access to moisture in the rooting zone (i.e. deeper root systems, altered root traits) to lessen potential damage. In contrast, soil drought acclimation tends to reduce stomatal sensitivity (e.g. Novick *et al*. 2016; Grossiord *et al*. 2018), making it unclear how combined temperature/VPD and soil droughts might alter stomatal responses in the long term.

Independent of stomatal acclimation, trees experience increasing stress during compound droughts, requiring simultaneous acclimation in the hydraulic vulnerability of conductive stem tissues (as commonly estimated with water potential inducing 50% loss of conductive surfaces, P50). Acclimation of P50 in response to soil drought has been documented in a few studies (Tomasella *et al*. 2017; Lemaire *et al*. 2021), highlighting the capacity of some species to adjust to water stress. Nevertheless, whether these shifts will be sufficient to compensate for the increase in stress levels remains unknown. Moreover, contrasting findings have also emerged, with both coniferous and broadleaf species showing little to no acclimation to changes in P50 (e.g. Limousin *et al*. 2022; Gauthey *et al*. 2024), suggesting that stem hydraulic acclimation is highly species‐ and context‐dependent. In contrast, leaf‐level hydraulic functions seem to be more plastic. Reported acclimation to elevated temperature and VPD in the presence or absence of soil drought include changes in physiological traits like reduced g_min_ (e.g. Duursma *et al*. 2019) or adjusted turgor loss point (e.g. Schönbeck *et al*. 2022; Weithmann *et al*. 2022), which can extend tree survival during compound droughts (Mekarni *et al*. 2024; Mas *et al*. 2024). To our knowledge, the acclimation of stem capacitance has yet to be documented, but studies comparing it across regions consistently report that trees in chronically drier and hotter climates tend to possess higher capacitance (e.g. Davey *et al*. 2023).

Moreover, compound droughts ultimately lead to an excess of excitation energy which cannot be safely dissipated in the photosystems when stomatal closure limits CO_2_ assimilation. Thus, acclimation of the photosynthetic reaction and metabolic processes will be decisive to withstand compound droughts (Gjindali & Johnson 2023). As pointed out above, secondary metabolites play an important acclimation role to drought, heat and excess energy, protecting photosynthetic process by counteracting ROS damage (Noctor *et al*. 2014). However, biochemical acclimation potential of different species is highly uncertain, although neglecting acclimation can result in significant errors in predicting photosynthetic performance (Fang *et al*. 2023). In this respect, volatile cues, specifically isoprene, are of particular interest as the can enhance leaf thermal tolerance of the emitting species as well as their neighbours (Singsaas, 2000). However, while isoprene emission may increase in some species and ecosystems, it is highly context‐dependent (Lantz *et al*. 2019).

Acclimation across hydraulic and physiological traits will undoubtedly shape tree resilience to future compound droughts. Yet, its effectiveness may depend even more on the capacity of structural traits to adapt to these intensifying challenges.

In general, previous work has highlighted a shift towards more conservative strategies, with reduced leaf area coupled with increased water acquisition through deeper roots, thereby supporting Darcy's law (McDowell & Allen 2015; Fig. 5). Field‐based studies with long‐term soil moisture manipulation have shown sparser canopies after prolonged drought exposure (e.g. Gauthey *et al*. 2023; Fatecha *et al*. 2024; Hesse *et al*. 2024), reflecting adjustments in leaf area and leaf‐to‐sapwood area ratio in response to soil moisture changes (e.g. Martínez‐Vilalta *et al*. 2009; Rosas *et al*. 2019; Anderegg *et al*. 2022), although only a few studies have identified the concurrent impacts of elevated temperature and VPD (e.g. Mas *et al*. 2024). A reduced leaf area lowers water demand and forces trees to rely more on sensible heat flux for cooling (e.g. Gauthey *et al*. 2023; Muller *et al*. 2023), which, together with adjustments in thermal tolerance, may help mitigate future thermal damage. Similarly, very few studies have reported evidence of acclimation of photosynthetic thermal tolerance (e.g. Zhu *et al*. 2018; Slot *et al*. 2021), with some suggesting that shifts in critical leaf temperature may be insufficient to overcome thermal stress during compound events (e.g. Kullberg & Feeley 2024). Increased investment into roots is also common, allowing deeper water extraction in long‐term soil droughts (Bachofen *et al*. 2024). However, the combined impact of high air temperature and VPD on rooting depth remains unclear. For instance, in a semi‐arid woodland exposed to chronic warming and soil drought, Grossiord *et al*. (2017) found that warming impairs the ability of some species to take up water from deeper water sources. Such acclimation might even be passed on to the next generation via epigenetic inheritance, as shown in a 20‐year irrigation study (Bose *et al*. 2020). Together, these physiological and morphological shifts may buffer against the cascading ecological disruptions caused by novel droughts, which would otherwise threaten ecosystem health and species interactions. Still, the time needed for such acclimation strategies to develop, and the extent of acclimation potential within and between species is largely unknown (Brodribb *et al*. 2020). Thus, whether leaf‐to‐tree level acclimation is sufficient to compensate or minimize the impacts of compound events also remains elusive.

## CONSEQUENCES FOR ECOSYSTEM PROCESSES

While leaf‐to‐tree level acclimation plays a crucial role in determining individual tree survival during compound droughts, it can also have cascading effects on ecosystem‐level processes, influencing water, carbon, and nutrient cycling across broader landscapes. Changes in canopy forest structure from acclimation (or direct stress impacts) can trigger changes in carbon and water fluxes in ecosystems through multiple feedback loops with biotic and abiotic factors. As pointed out above, canopy dieback, leaf shedding, and ultimately tree mortality significantly impacts forest microclimate (Zellweger *et al*. 2020; de Frenne *et al*. 2021), for example by reducing the protective role of dense canopies for the understorey (de Frenne *et al*. 2021; Werner *et al*. 2021). Canopy openings from dieback or sparser canopies enhance light penetration and heat in the understorey and soil surface (Anderegg *et al*. 2013a), increasing evaporative losses from the forest floor and thereby exacerbating water shortages (Zellweger *et al*. 2020). Drought‐induced canopy openings further alter understorey light conditions by increasing both irradiance and changes in light spectra, exposing vegetation to more intense and continuous light rather than transient sunflecks — factors that should be acknowledged as key drivers of forest ecosystem change. Thermophylization, that is, the shift to warmer‐adapted understorey species, has been associated with these forest microclimate changes in Europe (Zellweger *et al*. 2020). Moreover, these altered forest structures impact albedo and canopy reflectance, with strong feedback on latent and sensible heat flux (Anderegg *et al*. 2013c).

Generally, the diversity in tree hydraulic strategies among trees can help buffer forests against extreme drought impacts (Anderegg *et al*. 2018a; Grossiord 2020; Werner *et al*. 2021). Plant functional traits, particularly those related to water transport, play a crucial role in ecosystem resilience and land–atmosphere interactions during droughts (Anderegg *et al*. 2019). However, extreme droughts can alter competitive dynamics between neighbouring tree species (e.g. Haberstroh *et al*. 2021; Hackmann *et al*. 2026, this issue; Schmied *et al*. 2026, this issue). Beneficial interactions under mild drought (e.g. Pretzsch *et al*. 2013) can shift to enhanced competition under extreme drought (Grossiord *et al*. 2020; Haberstroh & Werner 2022). Additionally, compound droughts may change species composition, yielding unpredictable ecosystem‐scale responses due to interactions between community composition and physiological responses of individual species (Aguirre‐Gutiérrez *et al*. 2019). These shifts in plant–plant and plant–soil interactions and species composition can significantly alter ecosystem–atmosphere feedback, such as ecosystem CO_2_ exchange. For example, it was recently shown that drought legacy effects on gross primary productivity differed between mixed versus pure beech forests (Yu *et al*. 2022). Severe compound events can shift ecosystems towards their tipping points, which can induce a shift among dominant species and contribute to forest decline (Moore 2018; Dakos *et al*. 2019; Armstrong McKay *et al*. 2022; Haberstroh *et al*. 2022b). This has, for example, been observed following the 2018 hot drought in a pine forest on highly drained soils (Haberstroh *et al*. 2026, this issue). Furthermore, the stress response of ecosystems can result in transient increases in VOC emissions in response to heat and drought (e.g. Sindelarova *et al*. 2014). Under prolonged drought, even a cascading sequential increase of ecosystem‐scale VOC concentrations, from isoprene via monoterpenes to green leaf volatiles, has been observed, tracking drought severity (Werner *et al*. 2021). Subsequent mortality‐induced changes in dominant species with different emission blends as well as drought‐induced compositional changes in VOC emissions can induce VOC–climate feedbacks (Byron *et al*. 2022), through ozone, organic aerosol formation (Arneth *et al*. 2010; Guenther *et al*. 2012), as well as aerosol–radiation interaction and formation of cloud condensation nuclei (Makkonen *et al*. 2012; Pfannerstill *et al*. 2018).

Moreover, compound droughts facilitate forest disturbance from insect outbreaks, and significantly impact the integrity of forest ecosystems (Seidl *et al*. 2017; Pile *et al*. 2019), with bark beetle infestation of spruce being a most prominent recent example (Hart *et al*. 2017; Biedermann *et al*. 2019; Netherer *et al*. 2021). In contrast, pathogen outbreaks are more likely associated with warmer and wetter conditions (Seidl *et al*. 2017). Exceptional drought events, directly and in combination with biotic disturbances, often push temperate forests beyond their sustainability thresholds (Millar & Stephenson 2015; Senf & Seidl 2021). These biotic stresses, particularly herbivory, can induce significant VOC emissions (Holopainen & Gershenzon 2010; Faiola & Taipale 2020), for example, from these large‐scale insect outbreaks, potentially affecting secondary organic aerosol formation and the radiative properties of clouds (Holopainen *et al*. 2022).

Moreover, as discussed above (Section 4), the ecosystem sensitivity to VPD versus soil moisture is shaped by soil texture, which can determine the location of the hydraulic bottleneck on the soil–plant continuum (Carminati & Javaux 2024; Wankmüller *et al*. 2024). The high spatial heterogeneity in the impact of drought on forests further underlines the importance of accounting for small‐scale differences in soils and plant‐available water, together with neighbour density and canopy structure (e.g. Schmied *et al*. 2023), in order to adequately describe the spatial variability of drought‐affected trees.

Compound droughts can also strongly impact the belowground community structure of forests. For example, the abundance of soil mesofauna such as colembolla and mites was reduced after 8 years of recurrent summer drought (Lindberg *et al*. 2002), but largely recovered after 3 years, with mobile groups tending to recover more quickly (Lindberg & Bengtsson 2006). Among soil microorganisms, in particular, fungi and bacteria are thought to mediate the response of forest ecosystems to global change (Baldrian *et al*. 2023). Drought typically reduces soil microbial biomass and changes community structure by increasing the ratio of fungal to bacterial biomass (Wang *et al*. 2021; Baldrian *et al*. 2023). Under recurrent experimental summer drought, the composition of the ectomycorrhizal community was already affected during the first year of drought, with dissimilarity to control communities progressing in subsequent drought summers (Nickel *et al*. 2018). The composition and diversity of symbiotic endophytic and ectomycorrhizal fungi are closely related to tree vigour. Similarly, arbuscular mycorrhizal fungi increase the resilience of woody plants to recurrent drought, while bacterial communities have no clear effect (Barros *et al*. 2018; Anthony *et al*. 2024). Thus, responses of symbiotic microbial groups are expected to be important for plant resilience under changing climate and its legacy, suggesting that plants may be affected even if they do not experience the stress event themselves (Baldrian *et al*. 2023; Boyle *et al*. 2024).

## OUTLOOK

To continue advancing our understanding of plant responses to compound droughts, we need a multifaceted approach that integrates experimental, physiological and modelling perspectives (Werner *et al*. 2024). Experimental platforms simulating novel compound droughts, including recurrent drought events, are essential for disentangling the individual and interactive effects of climate stressors across spatial and temporal scales. Such platforms allow for a more precise assessment of drought legacies, particularly at the soil–root interface, where hydraulic properties play a crucial role in long‐term plant resilience, but are still poorly understood.

At the physiological level, a deeper understanding of the regulatory mechanisms governing the trade‐off between water loss, transpirational cooling, hydraulic processes, as well as carbon assimilation and allocation, into growth or defence is necessary. These processes underpin plant survival and ecosystem stability, yet their interactions remain insufficiently explored. Similarly, improving the mechanistic representation of temperature, VPD and soil drought interactions in climate–vegetation models is critical. Incorporating acclimation processes more explicitly into these models will enhance predictions of plant and ecosystem responses under future climate scenarios.

Finally, legacy effects and acclimation potential must be further explored, not only in terms of physiological responses but also considering phenological and structural adjustments. Understanding how past drought experiences shape future plant performance will provide key insights into ecosystem resilience and inform conservation and management strategies in the face of escalating climate extremes.

## AUTHOR CONTRIBUTIONS

CW, MB, TEEG and, CG conceptualized this study and wrote the manuscript. SH analysed data and provided description and data for Figs 1, 2, 3, and HV for Fig. 4. Data for Fig. 4 were provided by GL and DT. All authors read and endorsed the final version of the manuscript.

## Data Availability

Gridded data for Tmax, Tmin and AVP are available from the Climate Research Unit (CRU v. 4.08; University of East Anglia) and NCAS (Harris *et al*. 2014, 2020) and can be downloaded via the CEDA archive (https://data.ceda.ac.uk/badc/cru/data/cru_ts/cru_ts_4.08). Gridded data for SPEI6 are available from the global SPEI database (v. 2.9) (Beguería *et al*. 2010a, 2014; Vicente‐Serrano *et al*. 2010a) and can be downloaded via the global SPEI database (https://spei.csic.es/database.html). Shapefiles for the biomes in Fig. 1 can be downloaded from https://ecoregions.appspot.com/. The shapefile for Europe can be accessed at Natural Earth (https://www.naturalearthdata.com/).

## APPENDIX A

### Methodology for Fig. 1

Data for the analysis of minimum standardized precipitation evapotranspiration index (SPEI) were provided by the global SPEI database (v. 2.9) on a 6‐month basis (Beguería *et al*. 2010a, 2014; Vicente‐Serrano *et al*. 2010a), which is based on the CRU gridded Time Series (TS) v. 4.07. The data had a spatial resolution of 0.5°C and covered all land masses except Antarctica between 1901 and 2022 (Harris *et al*. 2020), while only the period 1961–2022 was further analysed. In February 2025, the global SPEI database (v. 2.10), which also covers the year 2023, potentially contained errors regarding the interpolation process and thus, the authors recommended using the global SPEI database (v. 2.9).

Data for analysis of maximum air temperature (*T*_max_) and vapour pressure deficit (VPD_max_) were provided by the Climatic Research Unit (CRU v. 4.08) (University of East Anglia) and NCAS (Harris *et al*. 2014, 2020) and downloaded via the CEDA archive. The included parameters from the CRU gridded Time Series (TS) v. 4.08 included monthly maximum temperature (T_max_; *tmx*), monthly minimum air temperature (T_min_; *tmn*) and monthly mean actual vapour pressure (AVP, *vap*). The data have a spatial resolution of 0.5°C and cover all land masses except Antarctica between 1901 and 2023 (Harris *et al*. 2020), while only the period 1961–2022 was further analysed in accordance with the available dataset for SPEI. All data were processed in R (v. 4.42) (R Core Team 2024) and read as netCDF files.

VPD (kPa) was calculated as (Allen *et al*. 1998; He *et al*. 2022):

(1)
VPD=SVP−AVP

where SVP is saturation vapour pressure (kPa) and AVP is actual vapour pressure (kPa). SVP (kPa) was calculated with Equation (2):

(2)
$$\mathrm{SVP}=\begin{array}{l} 0.5\times (0.611\times \exp\left(\frac{17.3\times T_{\min}}{T_{\min}+237.3}\right)+0.611\\ \times \exp\left(\frac{17.3\times T_{\max}}{T_{\max}+237.3}\right)) \end{array}$$

where *T*_min_ is minimum monthly air temperature (°C) and *T*_max_ is maximum monthly air temperature (°C). Ideally, SVP in Equation (2) should be calculated with daily data; however, He *et al*. (2022) argue that SVP calculated with daily and monthly air temperature data yields similar results.

All parameters retrieved from both datasets (*T*_max_, VPD, SPEI) were merged per year and month according to their longitude and latitude. For each grid cell, the maximum annual *T*_max_, maximum annual VPD and minimum annual SPEI were extracted. These values were averaged per year over all grid cells (global trends in Fig. 1A–C) and five biomes (Fig. 1D–I).

Data were separated into five biomes (boreal, temperate, Mediterranean, deserts & xeric shrublands, and tropical & subtropical) using the Ecoregions shapefile provided in Resolve (https://ecoregions.appspot.com/) (Dinerstein *et al*. 2017) revised for the version of Olson *et al*. (2001).

Here, it must be acknowledged that not all grid cells contain the same amount and time coverage of observations. While Harris *et al*. (2020) warn that regional and global climate trends assessed with CRU‐TS might contain artefacts, they argue that temperature is resilient to this problem because of its long correlation decay distance (CDD, 1200 km). Similarly, AVP has a CDD of 1000 km and is based on observations of temperature and temperature range (Harris *et al*. 2020). Additionally, we cross‐checked the presented trends for *T*_max_ computed with the CRU‐TS with the HadEX3 dataset for gridded land surface extremes (Dunn *et al*. 2024a) and excluded grid cells with <90% coverage between 1961 and 2018. Results clearly indicate that the patterns are consistent between datasets.

### Methodology for Fig. 2

Monthly data (VPD and SPEI) for Fig. 2 were downloaded and analysed as described in the Methodology for Fig. 2 and filtered for the Northern Hemisphere (latitude >0) and Europe (excluding Russia). To retrieve data for Europe, the *Country* shapefile from Natural Earth (Made with Natural Earth) was used. Data were filtered for the standard reference period of 1961–1990 and averaged per month. The monthly deviation of VPD and SPEI to the standard reference period of all months between 1991 and 2022 was further calculated (Fig. 2A,C). These deviations were averaged to demonstrate the mean annual deviation of VPD and SPEI per month between 1991 and 2022 from the standard reference period (Fig. 2B,D).

### Methodology for Fig. 4

We use land surface temperature (LST) from MODIS data. For each grid cell, we extracted maximum monthly temperatures using the MOD11A1 product, which provides daily land surface temperature from 2001 to 2021. Pixels of low quality, for example, contaminated by clouds or errors in the estimation of emissivity, were filtered using the product's quality band. Missing values were then gap‐filled with bilinear interpolation using the nearest available LST in space. Following the methodology of previous work (Duveiller *et al*. 2018; Guo *et al*. 2023) to approximate canopy temperature with LST, non‐vegetation pixels were first filtered out using a 1‐km vegetation pixel fraction map based on the ESA land cover map (Defourny 2019). We filtered the densely vegetated areas, excluding areas with a leaf area index <2, using the MODIS MCD15A3H product. Maps were transformed to 5‐km resolution, excluding pixels with NA values.

Per each year, the maximum monthly LST value was selected and used for the rest of the analysis. We calculated the trend per pixel from 2001 to 2021 based on a linear model and displayed the slope on a map. For specific areas (Western Mediterranean = long −4 to 1°, lat 40 to 43°; Temperate = long 8 to 13°, lat 49 to 53°; Boreal = long 25 to 30°, lat 61 to 64°; and Eastern Mediterranean = long 30 to 35°, lat 38 to 41°) we calculated the maximum LST per area and displayed results in a regression. We also mapped the average maximum LST from 2001 to 2021, and the maximum LST in 2003 and 2018.
